# Rotation system reshapes soil profile environmental gradients to reorganize bacterial communities and decouple potential nitrogen cycling functions

**DOI:** 10.3389/fmicb.2026.1979930

**Published:** 2026-10-14

**Authors:** Wenping Zhang, Qinyue Chen, Yunguo Li, Yan Chen, Guoyi Yuan, Yu Du, Bing Wang, Xinran Sun, Nuo Shi, Xuanquan Zhu, Meng Jia, Yuxiang Bai, Xiaopeng Deng, Peng Zhou, Ge Wang, Na Wang

**Affiliations:** 1 College of Tobacco Science, Yunnan Agricultural University, Kunming, Yunnan, China; 2 The Key Laboratory of Agricultural Microbiome of Yunnan Province, Kunming, Yunnan, China; 3 Yuxi Tobacco Company Yuanjiang County Branch, Yuxi, Yunnan, China; 4 Honghe Hani and Yi Autonomous Prefecture Academy of Science and Technology, Honghe, Yunnan, China; 5 Yunnan Provincial Institute of Tobacco and Agricultural Sciences, Kunming, Yunnan, China

**Keywords:** cropping systems, microbial co-occurrence network, nitrogen cycle, soil ecological functions, soil microbial communities, soil profile

## Abstract

**Inroduction:**

Cropping systems reshape soil profile environmental gradients, thereby driving the vertical reorganization of bacterial communities and regulating the coupling between microbial communities and predicted nitrogen-cycling (N-cycling) potential.

**Methods:**

This study integrated soil chemical analyses, 16S rRNA gene sequencing, co-occurrence network analysis, PICRUSt2-based functional prediction, and structural equation modeling (SEM) across three long-term cropping systems: unclaimed land (U), tobacco–maize rotation (R), and continuous tobacco cropping (C), sampled at three soil depths (0–20, 20–40, and 40–60 cm; *n* = 3).

**Results:**

Environmental drivers exhibited distinct vertical shifts, with readily available nutrients (AN, AK, and AP) dominating surface soils (0–20 cm), microbial biomass (MBC and MBN) governing subsurface soils (20–40 cm), and total nutrient pools (OM, TN, and TP) becoming increasingly important in deeper soils (40–60 cm). Rotation buffered vertical environmental changes, increased bacterial richness in the 0–40 cm profile, enriched nitrifying and nitrogen-fixing taxa (e.g., Nitrospira, Sphingomonas, and MND1), and maintained higher predicted N-cycling potential throughout the soil profile, accompanied by enhanced network modularity and robustness. In contrast, continuous cropping reduced surface soil pH, OM, MBC, and MBN (with MBN declining by>60%), promoted stress-associated taxa (e.g., Bacillus and Lactobacillus), and weakened network connectivity and robustness, particularly at 20–40 cm where network fragmentation was most evident. SEM revealed a stronger statistical coupling among soil environment, bacterial communities, microbial networks, and predicted N-cycling potential under rotation (GOF > 0.7), whereas continuous cropping showed weakened associations among these components.

**Discussion:**

Collectively, these findings demonstrate that crop rotation promotes the stability of soil microbial ecological functions by preserving environmental gradient continuity and enhancing network resilience, whereas continuous cropping disrupts microbial network organization and weakens the linkage between resource availability and predicted functional potential. This study highlights the importance of maintaining multiscale environmental and microbial connectivity for improving the sustainability of agricultural soil ecosystems.

## 1 Introduction

Soil microorganisms are fundamental regulators of terrestrial biogeochemical cycles, controlling organic matter decomposition, nutrient transformation, and ecosystem functioning (Sokol et al., 2022; Dalléac et al., 2026; Iqbal et al., 2025). Among these processes, N-cycling directly links soil fertility, plant productivity, and ecosystem stability, and is largely governed by the composition and functional potential of microbial communities (Mattoo and Suman, 2023). Key nitrogen transformation processes, including nitrification, denitrification, and biological nitrogen fixation, are mediated by specialized microbial groups (Chen et al., 2022; Hong et al., 2022; Yu et al., 2023). Therefore, understanding how agricultural management practices regulate microbial community assembly and associated N-cycling potential is essential for predicting soil ecological sustainability (Dou et al., 2023; Shu et al., 2024).

Cropping systems represent a major anthropogenic driver of soil microbial ecological processes by altering resource availability, soil physicochemical conditions, and plant-derived inputs (Domnariu et al., 2025; Wang Y. et al., 2025). Continuous cropping frequently induces soil acidification, nutrient imbalance, and microbial community degradation (Li L. et al., 2025; Gao et al., 2024; Huang et al., 2025), whereas crop rotation can improve soil ecological functioning by diversifying organic inputs, modifying rhizosphere processes, and enhancing N-cycling (Li C. et al., 2025; Chen P. et al., 2025). However, previous studies have mainly focused on surface soils (0–20 cm) and community-level patterns, providing limited mechanistic understanding of how cropping systems regulate microbial functional potential throughout the entire soil profile. In particular, how rotation and continuous cropping reshape bacterial communities through vertical environmental filtering and microbial interaction networks, and how these changes influence the coupling between microbial communities and predicted N-cycling potential, remain poorly understood.

Soil profiles constitute vertically structured ecological gradients where pH, organic matter availability, oxygen conditions, and nutrient pools progressively change with depth, creating distinct microbial niches and assembly processes (Dove et al., 2021; Wang L. et al., 2025; Tian et al., 2023; Zhu et al., 2025). Increasing evidence indicates that subsurface and deep soils contribute substantially to nitrogen storage, long-term N-cycling, and ecosystem stability (Peng et al., 2025; Wang M. M. et al., 2025). Nevertheless, whether cropping systems regulate microbial ecological processes across the entire soil profile remains unclear. Specifically, the relationships among soil environmental gradients, bacterial community organization, ecological network characteristics, and predicted N-cycling potential across different soil depths have not been integrated into a unified framework. This knowledge gap limits our ability to predict how agricultural management practices influence the stability and sustainability of soil microbial functions.

To address these gaps, we developed a profile-scale framework based on environmental filtering theory and microbial community assembly principles. We hypothesized that: (i) cropping systems reshape vertical environmental gradients, thereby driving depth-dependent reorganization of bacterial communities, with rotation maintaining greater environmental continuity than continuous cropping; (ii) rotation strengthens the coupling among soil properties, microbial communities, ecological networks, and predicted N-cycling potential, whereas continuous cropping weakens these relationships; and (iii) the dominant environmental drivers of microbial community assembly shift along the soil profile, with available nutrients dominating surface layers, microbial biomass controlling subsurface layers, and total nutrient pools becoming increasingly important in deeper soils.

To test these hypotheses, we investigated a long-term flue-cured tobacco field in Yunnan under three cropping systems: unclaimed land (U), continuous tobacco cropping (C), and tobacco–maize rotation (R). Soil samples were collected from three depths (0–20, 20–40, and 40–60 cm) to characterize changes in soil physicochemical properties, bacterial community structure, co-occurrence network topology, and predicted N-cycling potential using PICRUSt2. Specifically, this study aimed to: (i) characterize the vertical responses of soil environmental gradients and bacterial communities under different cropping systems; (ii) identify depth-dependent environmental drivers regulating microbial community assembly; and (iii) elucidate the statistical relationships among environmental conditions, bacterial community organization, network structure, and predicted N-cycling potential through structural equation modeling. By integrating soil chemical analyses, 16S rRNA gene sequencing, network analysis, functional prediction, and multivariate modeling, this study provides mechanistic insights into how cropping systems regulate soil microbial ecological processes across soil profiles.

## 2 Materials and methods

### 2.1 Overview of the experiment

The experimental site was located at the Yuxi Yanhe Base of Yunnan Tobacco Science Academy (24°23′63″N, 102°49′98″E), at an elevation of 1,635 m in a subtropical humid monsoon climate zone. The mean annual temperature ranges from 17.4 to 23.8 °C, annual precipitation from 670.8 to 1,500.2 mm, and the frost-free period from 146.2 to 168.3 days. The soil is classified as Ferric Argosols according to the Chinese Soil Taxonomy, with a silty loam texture. The initial soil physicochemical properties were as follows: pH 6.72, organic matter (OM) 16.24 g kg^−1^, total nitrogen (TN) 0.97 g kg^−1^, total phosphorus (TP) 0.79 g kg^−1^, total potassium (TK) 4.96 g kg^−1^, available nitrogen (AN) 67.53 mg kg^−1^, available phosphorus (AP) 48.37 mg kg^−1^, and available potassium (AK) 175.01 mg kg^−1^. The experiment was conducted using a randomized complete block design, divided into three long-term fixed cropping systems: (1) unclaimed land (U); (2) continuous tobacco cropping (C); and (3) tobacco-maize rotation (R). Each treatment included three spatially independent replicate plots (each 20 m^2^), totaling nine plots. A 1-meter-wide buffer zone was established between adjacent plots to minimize edge effects. Except for the cropping system, all agronomic practices were identical across treatments, and fertilization was uniformly managed (Table 1).

**Table 1:** Crop cultivation and fertilizer management in the experimental field from 2011 to 2021.

Cropping system	Unclaimed land (U)	Continuous tobacco cropping (C)	Tobacco-maize rotation (R)
Variety	Yunyan 87	Yunyan 87	Tobacco: Yunyan 87; Maize: Huaxing Dan No. 7
Growing period	April–May	April–May	Maize from March to April, Tobacco from April to May
Harvest period	August–September	August–September	Maize from July to August, Tobacco from August to September
Annual fertilizer application: base fertilizer	Tobacco-specific compound fertilizer (N:P:K ratio = 15:8:20) at 750–800 kg/hm^2^	Same as the unclaimed land	Maize season: Urea (N ≥ 46%) 150 kg/hm^2^; Tobacco season: Same as the unclaimed land
Annual fertilizer application: Top dressing	None	None	Maize season: Maize-specific compound fertilizer (N:P:K ratio = 29:13:10) at 600–700 kg/hm^2^; Tobacco season: None

All three treatments planted only one main crop per year, with no subsequent secondary cropping.

### 2.2 Soil sample collection

Soil sampling was conducted on August 15, 2021 (during the mature stage of flue-cured tobacco), with no precipitation recorded in the three days prior. Within each replicate plot of treatment, five sampling points were evenly distributed using the “five-point sampling method.” Surface litter, moss, and visible plant residues were gently removed from each sampling point. A stainless steel soil auger with an inner diameter of 5 cm (previously disinfected with 75% alcohol) was used to collect undisturbed soil cores from three depth layers (0–20 cm, 20–40 cm, and 40–60 cm) at each point. After completing one layer, the soil core was carefully extracted, and the inner wall of the auger was wiped with fresh soil from the next deeper layer before drilling into the subsequent depth, thus preventing contamination from upper-layer soil. Once all three layers at a given point were collected, the auger was cleaned with 75% alcohol-soaked cotton balls, air-dried, and then moved to the next sampling point within the same plot for further sampling. The five soil cores collected from the same plot, same soil layer, and five sampling points were thoroughly mixed. Visible stones (>2 mm), roots, and animal remains were removed, and the sample was reduced to approximately 2.0 kg using the quartering method. The sample was placed in sterile polyethylene self-sealing bags and immediately labeled. Each composite sample was divided into three portions according to analytical requirements: one portion was placed in sterile cryogenic tubes, rapidly frozen in liquid nitrogen, and stored at −80 °C for microbial DNA analysis; another portion was air-dried and sieved through a 2 mm mesh for soil chemical property testing; the third portion was placed in an insulated cooler with ice packs and transported back to the laboratory within 2 h, then stored at 4 °C for soil chemical analysis.

### 2.3 Soil chemical property analysis

Soil pH was measured using the electrode method at a soil-to-water ratio of 1:2.5 (w/v) (Wang and Han, 2022). OM was determined by potassium dichromate oxidation (Chen et al., 2024), TN by the Kjeldahl method (Guo et al., 2021), TP by acid digestion followed by molybdenum-antimony colorimetry (Wang et al., 2022), and TK by flame photometry (Yu et al., 2026). AN was determined by the alkaline hydrolysis diffusion method (Xiang et al., 2022), AP by NaHCO₃ extraction followed by molybdenum-antimony colorimetry (Li et al., 2021b), and AK by ammonium acetate extraction followed by flame photometry (Gu et al., 2021). NH₄^+^-N was determined by the 10% NaCl extraction-distillation method, and NO₃^−^-N by the phenol disulfonic acid colorimetric method (Li et al., 2021a). MBC and MBN were determined using the chloroform fumigation-K₂SO₄ extraction method (GB/T 39228–2020). Each physical and chemical parameter was independently measured for each biological replicate sample (*n* = 3), and the results are expressed as “mean ± standard deviation.”

### 2.4 Soil microbial DNA extraction and sequencing

Total DNA was extracted from 1.0 g of fresh soil using the PowerSoil DNA Extraction Kit (QIAGEN, USA). DNA concentration and purity were determined by UV spectrophotometry, and DNA integrity was assessed by 0.8% agarose gel electrophoresis. DNA samples were stored at −20 °C until further analysis. The V3-V4 region of the bacterial 16S rRNA gene was amplified by PCR using primers 341F (CCTACGGGNGGCWGCAG) and 806R (GGACTACHVGGGTATCTAAT). PCR products were verified by 2% agarose gel electrophoresis, excised, purified, and used to construct sequencing libraries for high-throughput sequencing on the Illumina MiSeq platform.

### 2.5 Bioinformatics analysis

Raw reads were quality-filtered using FASTP and merged using FLASH to generate high-quality sequences (clean tags). Chimeric sequences were removed using Usearch, and operational taxonomic units (OTUs) were clustered at 97% sequence similarity using UPARSE (v7.0.1001). Representative sequences were taxonomically assigned using the RDP Classifier based on the Naïve Bayesian algorithm.

### 2.6 Data analysis

Soil physicochemical data were analyzed using Excel 2019 and SPSS 26.0. Differences among treatments were evaluated by one-way analysis of variance (ANOVA) followed by Duncan’s multiple range test. Microbial α-diversity, principal coordinate analysis (PCoA) based on Bray-Curtis distances, microbial community composition, linear discriminant analysis effect size (LEfSe), and redundancy analysis (RDA) were performed using the OmicShare platform.^1^ Functional prediction was conducted using the Metware Cloud platform.^2^ Random forest analysis was performed in R using the readxl, ggplot2, reshape2, and rfPermute packages. Co-occurrence network construction and topological analyses were conducted in R using the tidyverse, igraph, Hmisc, and related packages. Prior to Spearman correlation analysis, the operational taxonomic unit (OTU) table was filtered to remove low-abundance OTUs with a mean absolute abundance < 30 across all samples, thereby minimizing the potential interference of rare taxa in network inference. Based on the filtered OTU relative abundance matrix, Spearman correlation coefficients and corresponding *p*-values between OTUs were calculated using the Hmisc package, and only significant correlations with |r| > 0.85 and *p* < 0.05 were retained for network construction. Network topological parameters, including node number, edge number, average degree, modularity, vulnerability, and robustness, were calculated using the igraph package. To evaluate differences in network properties among treatments, sub-networks were extracted based on OTUs present in each individual sample, and topological metrics were calculated; one-way ANOVA or Kruskal-Wallis tests were then used to compare these metrics among treatments. Structural equation modeling was performed in R using the plspm, readr, caret, dplyr, and devtools packages. Figures illustrating the proposed mechanism were prepared using Adobe Illustrator 2024 and WPS Office.

All statistical analyses (including ANOVA, LEfSe, and network analysis) were performed using samples from three replicate plots as biological replicates (*n* = 3). Technical replicates of instrument readings were averaged prior to statistical testing and were not treated as independent samples.

## 3 Results

### 3.1 Profile response of soil chemical properties

Two-way ANOVA (Table 2, *p* < 0.05) revealed significant interactions between cropping system and soil depth for most soil physicochemical properties, indicating that cropping system effects on soil environments varied across the soil profile. R maintained relatively stable nutrient conditions throughout the 0–60 cm profile, with no significant reductions in OM, TN, AN, or NH₄^+^-N. Moreover, R significantly increased AP, AK, and NO₃^−^-N concentrations in the 0–20 cm layer, suggesting enhanced nutrient availability in surface soils while preserving overall nutrient stability across the profile. In contrast, C substantially altered surface soil properties, resulting in significant decreases in pH, OM, nitrogen-related nutrients, MBC, and MBN, with MBN declining by more than 60%. Meanwhile, C increased surface AP, AK, and NO₃^−^-N concentrations, indicating nutrient accumulation in the topsoil despite reduced microbial biomass and biological nutrient availability. Collectively, these results demonstrate that long-term cropping systems reshape soil environmental gradients and increase vertical heterogeneity in soil physicochemical properties.

**Table 2:** Soil chemical properties at different soil layers under various cropping systems.

Soil variables	0–20 cm			20–40 cm			40–60 cm			Two-way ANOVA		
	U	R	C	U	R	C	U	R	C	Cropping systems	Soil depth	C × S
pH	7.78 ± 0.06a	7.55 ± 0.10b	6.82 ± 0.04c	7.63 ± 0.05a	7.31 ± 0.04b	7.09 ± 0.07c	7.68 ± 0.06a	7.43 ± 0.11b	7.27 ± 0.07b	***	**	***
OM (g/kg)	15.6 ± 0.60a	14.5 ± 0.60a	12.0 ± 0.60b	11.81 ± 0.60a	11.77 ± 0.50a	10.20 ± 0.50b	9.88 ± 0.50a	9.55 ± 0.50ab	8.62 ± 0.40b	***	***	*
TN (g/kg)	0.90 ± 0.04a	0.83 ± 0.05a	0.71 ± 0.05b	0.72 ± 0.05a	0.65 ± 0.05ab	0.57 ± 0.04b	0.67 ± 0.04a	0.60 ± 0.04a	0.46 ± 0.04b	***	***	ns
TP (g/kg)	1.01 ± 0.01b	1.15 ± 0.05a	1.17 ± 0.07a	0.85 ± 0.06b	1.03 ± 0.06a	1.04 ± 0.05a	0.97 ± 0.02a	1.00 ± 0.02a	0.98 ± 0.06a	***	***	*
TK (g/kg)	6.81 ± 0.09a	6.20 ± 0.15b	6.88 ± 0.10a	6.80 ± 0.08b	7.40 ± 0.08a	6.89 ± 0.07b	7.53 ± 0.11a	7.08 ± 0.07b	7.28 ± 0.08b	**	***	***
AN (mg/kg)	73.74 ± 1.80a	79.31 ± 2.30a	64.35 ± 2.70b	60.23 ± 2.30ab	62.36 ± 1.70a	55.95 ± 1.70b	47.88 ± 1.35a	46.10 ± 1.30ab	44.88 ± 0.70b	***	***	***
AP (mg/kg)	42.20 ± 2.10b	54.09 ± 1.65a	57.61 ± 1.10a	41.69 ± 1.57b	42.11 ± 0.50b	46.37 ± 1.48a	36.34 ± 1.71a	37.81 ± 1.31a	38.54 ± 1.28a	***	***	***
AK (mg/kg)	50.10 ± 0.98c	122.47 ± 6.00b	246.93 ± 6.83a	50.09 ± 1.98c	64.56 ± 2.98b	87.72 ± 3.94a	50.09 ± 2.05b	52.98 ± 3.01b	142.73 ± 4.99a	***	***	***
NO3-N (mg/kg)	20.25 ± 0.77c	34.34 ± 1.28b	44.35 ± 1.71a	18.87 ± 0.93b	19.85 ± 0.92ab	21.36 ± 0.86a	15.04 ± 0.89a	15.30 ± 0.70a	15.07 ± 0.89a	***	***	***
NH4+-N (mg/kg)	2.19 ± 0.07a	2.03 ± 0.08a	1.02 ± 0.10b	0.92 ± 0.10a	0.86 ± 0.08a	0.81 ± 0.10a	0.72 ± 0.10a	0.61 ± 0.10a	0.54 ± 0.10a	***	***	***
MBC (mg/kg)	18.18 ± 0.58a	16.00 ± 0.70b	12.00 ± 0.90c	10.50 ± 0.87a	9.00 ± 0.80ab	7.50 ± 0.60b	6.00 ± 0.60a	5.00 ± 0.70ab	3.80 ± 0.40b	***	***	**
MBN (mg/kg)	2.60 ± 0.16a	2.00 ± 0.26b	1.00 ± 0.19c	1.50 ± 0.24a	1.20 ± 0.18ab	0.75 ± 0.11b	0.90 ± 0.10a	0.70 ± 0.10ab	0.55 ± 0.05b	***	***	***

C, Cropping systems; S, Soil depth. Different lowercase letters indicate significant differences (*p* < 0.05) among different soil layers under the same cropping system. ns, not significant; *p < 0.05; **p < 0.01; ***p < 0.001; All data are expressed as mean ± standard error (mean ± SE).

### 3.2 Bacterial community diversity and structural spatial reorganization

Bacterial α-diversity (Figure 1A) was primarily regulated by cropping system, whereas soil depth exhibited a relatively weaker effect. R significantly increased bacterial richness in the 0–40 cm soil layer, while no significant differences were detected between C and U. β-diversity analysis based on Bray–Curtis dissimilarity (Figure 1B) revealed that bacterial community structure was significantly affected by cropping system, soil depth, and their interaction (*p* < 0.001). Community dissimilarity among cropping systems was greatest in the surface layer (0–20 cm) and progressively decreased with increasing soil depth. The bacterial communities in the 40–60 cm layer showed greater similarity across cropping systems, indicating enhanced environmental filtering and convergence of microbial community composition in deeper soils. The bacterial community was dominated by three major phyla, Proteobacteria, Firmicutes, and Acidobacteria (Figures 1C–H). Compared with other treatments, R significantly enriched several taxa associated with nitrogen transformation processes, including Nitrospira, Sphingomonas, and MND1. In contrast, C promoted the enrichment of stress-adapted and opportunistic taxa, such as Bacillus and Lactobacillus. Furthermore, Thaumarchaeota exhibited higher relative abundance under both agricultural management systems (R and C) compared with U, suggesting that cultivation altered the distribution of microbial groups potentially involved in nitrogen transformation. Collectively, these results indicate that cropping systems induced depth-dependent shifts in bacterial community diversity and composition, with rotation promoting a more functionally favorable microbial assemblage and continuous cropping selecting for stress-associated communities.

**Figure 1:**
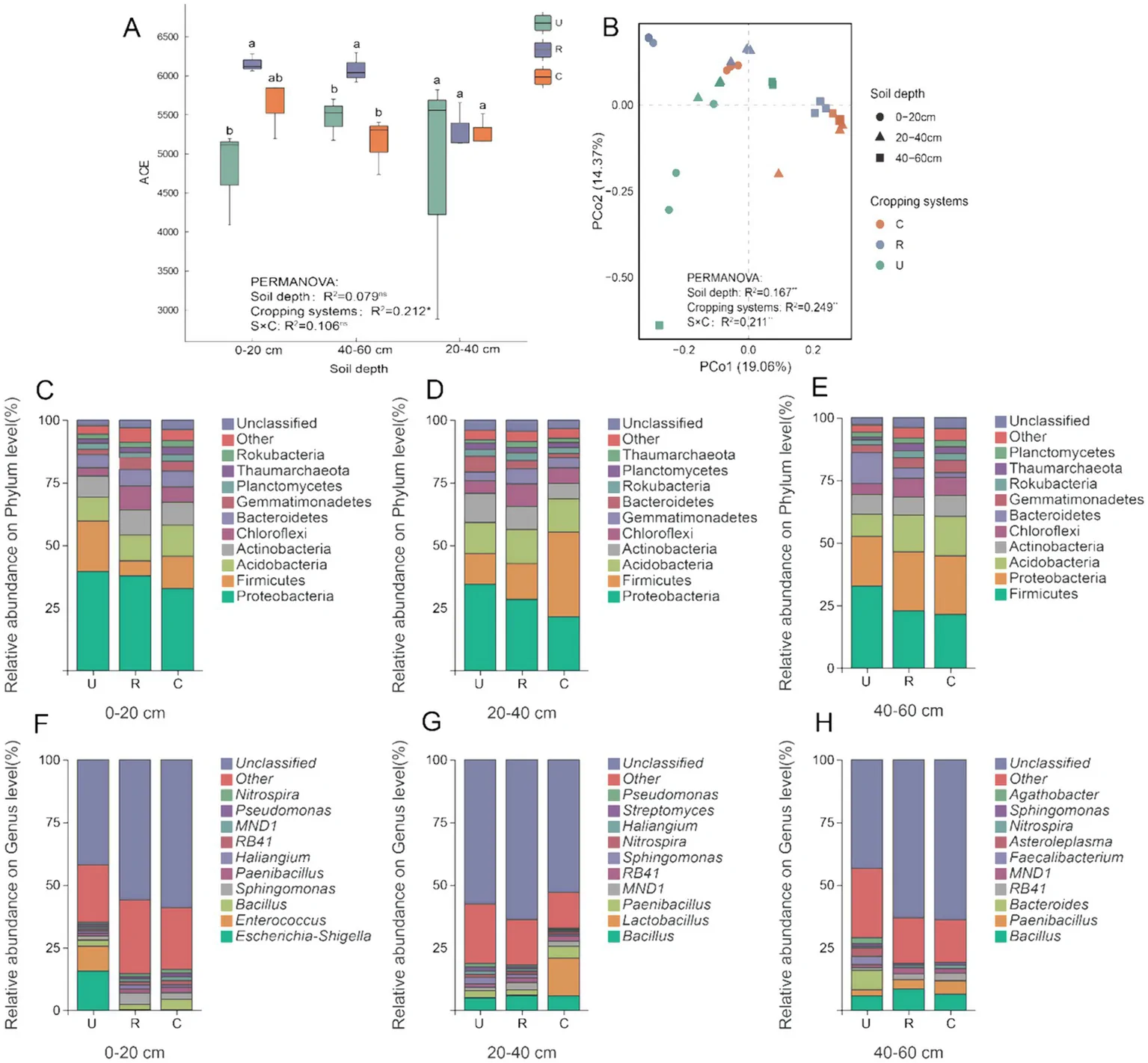
Soil bacterial community diversity and composition under different tillage systems and soil depths. **(A)** Soil bacterial α-diversity (ACE index), different lowercase letters indicate significant differences (*p* < 0.05) among different tillage systems at the same soil depth. **(B)** Principal coordinate analysis (PCoA) of bacterial community β-diversity. **(C–E)** Bacterial community composition at the phylum level across different soil layers. **(F–H)** Bacterial community composition at the genus level across different soil layers. U, Unclaimed land; R, Tobacco-maize rotation; C, Continuous tobacco cropping.

### 3.3 Key environmental factors in community assembly

Random forest (Figures 2A–C) and redundancy analysis (RDA; Figures 2D–F) revealed distinct shifts in the environmental factors associated with bacterial community assembly across soil depths. In the 0–20 cm layer, readily available nutrients, including AN, AK, AP, and TK, together with soil pH, showed strong associations with bacterial community variation, reflecting the pronounced influence of surface nutrient conditions under different cropping systems. In the 20–40 cm layer, the importance of environmental predictors shifted toward pH, MBC and MBN, indicating an increased contribution of microbial resource status to community organization in the subsurface soil. In the 40–60 cm layer, bacterial community composition was mainly associated with OM, TN, and TP, suggesting stronger constraints imposed by relatively stable nutrient pools in deeper soil layers. Collectively, these results demonstrate a depth-dependent transition in the factors associated with bacterial community assembly, from readily available nutrient conditions in surface soils to microbial biomass-related and total nutrient-related constraints in deeper soil layers.

**Figure 2:**
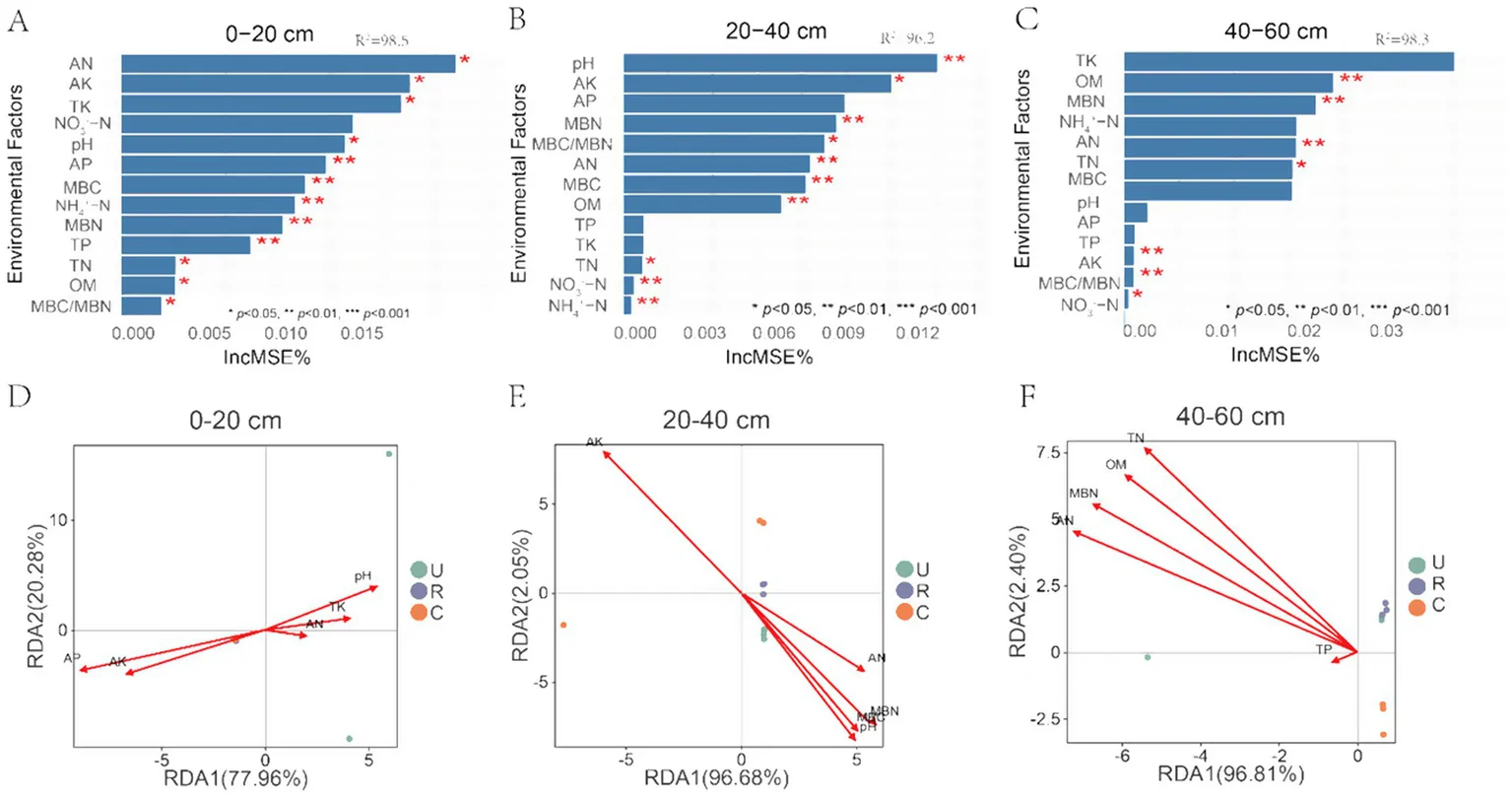
Effects of soil physicochemical properties on bacterial communities at different soil depths. A-C show the ranking of environmental factors’ influence on bacterial communities (random forest, IncMSE%) and significance test results in the 0–20 cm **(A)**, 20–40 cm **(B)**, and 40–60 cm **(C)** soil layers, respectively. *, *p* < 0.05; **, *p* < 0.01; ***, *p* < 0.001. **(D–F)** present redundancy analysis (RDA) of bacterial communities and soil physicochemical properties in the 0–20 cm **(D)**, 20–40 cm **(E)**, and 40–60 cm **(F)** soil layers, respectively.

### 3.4 Nitrogen-cycle functional differentiation

LEfSe (Supplementary Figures S1A–C) and PICRUSt2 analyses (Supplementary Figure S1D) revealed depth-dependent variation in predicted nitrogen-cycling functions among cropping systems. The number of differentially abundant taxa identified by LEfSe gradually decreased with increasing soil depth, indicating a reduced differentiation of bacterial communities among treatments in deeper soil layers. Compared with U and C, R showed higher relative abundances of microbial taxa and predicted functional pathways associated with nitrification, nitrogen fixation, and other nitrogen transformation processes, resulting in consistently higher predicted N-cycling potential across the soil profile. In contrast, C exhibited altered distributions of predicted nitrogen-related functional profiles and lower predicted potential for several nitrogen transformation processes. The U treatment showed intermediate levels of predicted N-cycling potential, reflecting the influence of natural soil conditions without long-term agricultural inputs. Collectively, these results demonstrate that cropping systems differentially shaped the distribution of bacterial taxa and predicted nitrogen-cycling functional profiles along the soil profile.

### 3.5 Spatial reorganization of microbial co-occurrence network structure

Soil depth was the dominant factor associated with variation in microbial co-occurrence network structure (*p* < 0.001). With increasing soil depth, network complexity, connectivity, and topological robustness decreased significantly, whereas modularity increased, revealing a transition from highly connected surface networks to more compartmentalized networks in deeper soil layers. Cropping systems mainly affected network properties within the 0–40 cm soil layer. Compared with U and C, R maintained higher network connectivity, complexity, and modularity, indicating a more integrated microbial interaction structure under rotation management. In contrast, C markedly reduced network connectivity and robustness, particularly in the 20–40 cm layer where network simplification was most pronounced. In the 40–60 cm layer, network topological characteristics showed greater similarity among cropping systems, suggesting that depth-related environmental constraints increasingly shaped microbial network organization. Overall, these results demonstrate that soil depth established the primary spatial framework of microbial co-occurrence networks, while cropping systems modified network organization mainly in surface and subsurface soils (Figure 3).

**Figure 3:**
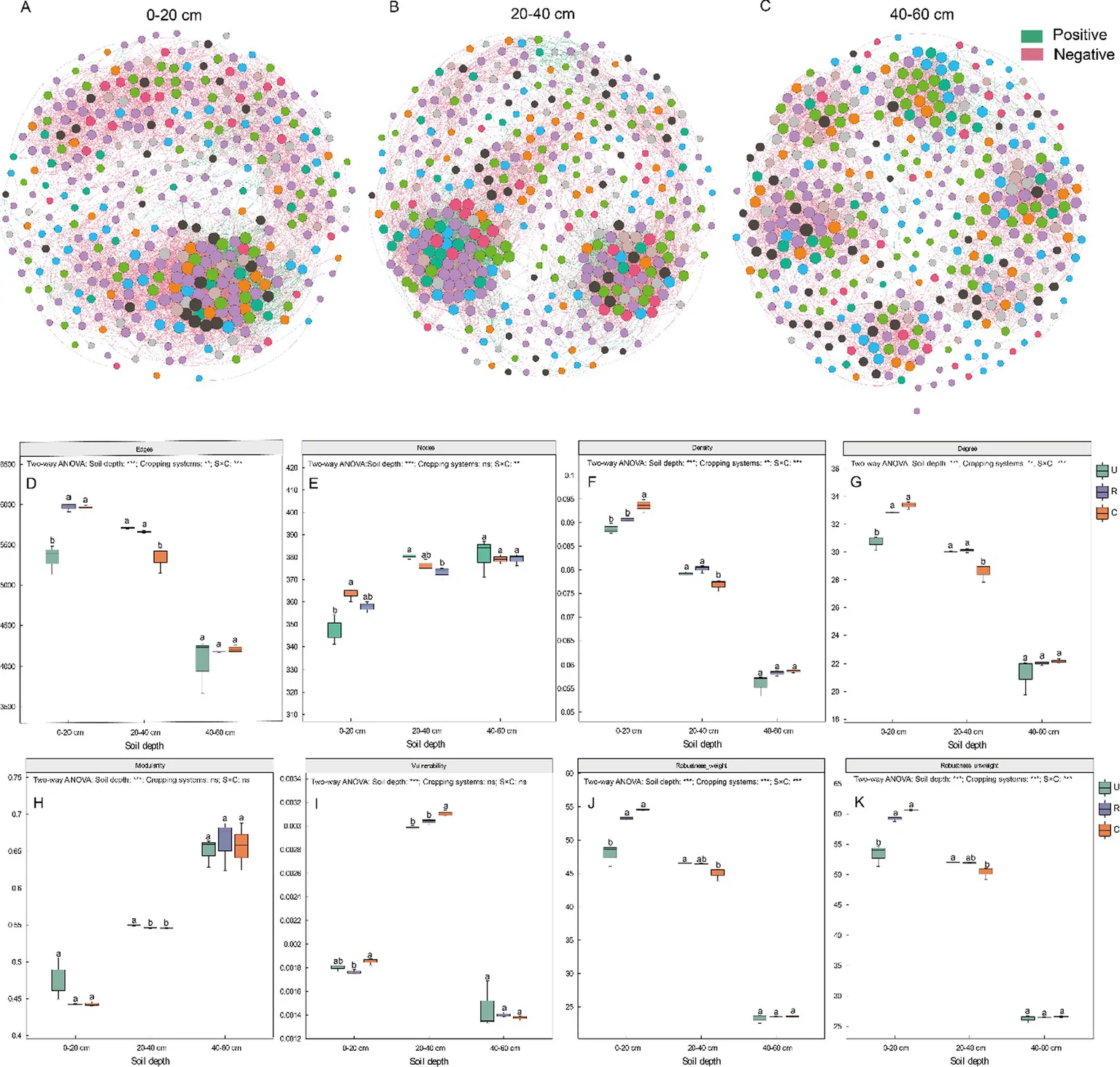
Co-occurrence network structure and stability characteristics of soil bacterial communities under different cropping systems and soil depths. **(A–C)** Soil bacterial co-occurrence networks constructed based on Spearman correlation at 0–20 cm **(A)**, 20–40 cm **(B)**, and 40–60 cm **(C)** soil depths. Nodes represent bacterial OTU-level taxonomic units, and edges indicate significant correlations between OTUs (*p* < 0.05, |r| > 0.85); red edges denote positive correlations, green edges negative correlations. **(D–K)** Differences in core topological properties of co-occurrence networks across soil depths and cropping systems: number of nodes **(D)**, number of edges **(E)**, network density **(F)**, average degree **(G)**, modularity index **(H)**, network vulnerability **(I)**, weighted robustness **(J)**, and unweighted robustness **(K)**. U, Unclaimed land; R, Tobacco-maize rotation; C, Continuous tobacco cropping. Results of two-way ANOVA are indicated above each subplot (ns, *p* > 0.05; *, *p* < 0.05; **, *p* < 0.01; ***, *p* < 0.001). Different lowercase letters on box plots indicate significant differences among treatments within the same soil layer (*p* < 0.05). C, Cropping systems. S, Soil depth.

### 3.6 Structural equation modeling comprehensive analysis

SEM revealed distinct association patterns among cropping systems based on cross-sectional observations. In the unclaimed land treatment (U; Figure 4A), soil chemical properties showed a negative association with predicted N-cycling potential, whereas microbial diversity was positively associated with bacterial community assembly but negatively associated with predicted functional potential within the inferred model. In the tobacco–maize rotation treatment (R; Figure 4B), soil chemical properties exhibited positive associations with predicted N-cycling potential through an inferred “soil environment–microbial community–network–function” pathway, indicating stronger statistical coupling among these components. In contrast, continuous tobacco cropping (C; Figure 4C) showed weakened associations among soil properties, microbial communities, network structure, and predicted N-cycling potential, suggesting a reduced statistical coupling pattern under long-term monocropping. Collectively, SEM indicated that cropping systems were associated with variation in predicted N-cycling potential through their relationships with soil environmental conditions and microbial network characteristics. However, these inferred pathways represent statistical associations rather than causal relationships and require further validation using experimental approaches.

**Figure 4:**
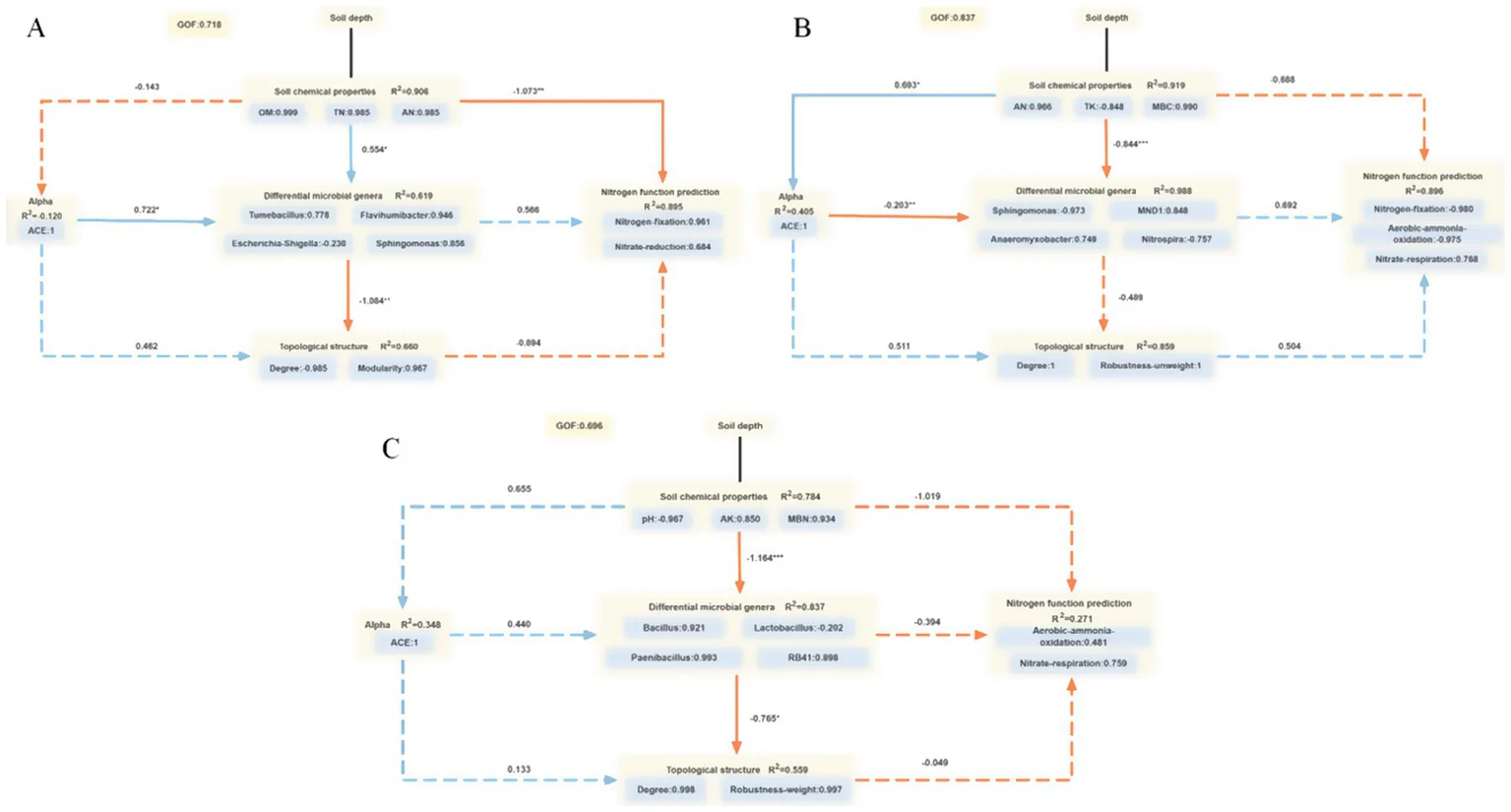
Driving pathways of soil bacterial community diversity, key taxa, and N-cycling functions under different cropping systems. **(A–C)** Structural equation models illustrating the relationships among variables under unclaimed land, tobacco–maize rotation, and continuous tobacco cropping, respectively. The models were evaluated using the goodness of fit (GOF) index, where GOF > 0.7 indicates excellent fit, 0.5–0.7 indicates good fit, and 0.3–0.5 indicates acceptable fit. Blue values on the box between measured variables and their corresponding latent modules represent indicator loadings, and R^2^ values, representing the explanatory power for each endogenous module, are shown in the figure. GOF indicates the model fit index. Orange solid arrows indicate significant negative correlations, orange dashed arrows indicate non-significant negative correlations, blue solid arrows indicate significant positive correlations, and blue dashed arrows indicate non-significant positive correlations. Values adjacent to arrows are standardized path coefficients; ns, *p* > 0.05; *, *p* < 0.05; **, *p* < 0.01; ***, *p* < 0.001.

## 4 Discussion

### 4.1 Hierarchical filtering mechanism driven by environmental gradients in the soil profile for microbial assembly

This study demonstrates that cropping systems interact with soil depth to regulate soil chemical properties, bacterial community composition, and microbial network characteristics, indicating that the ecological effects of agricultural management are vertically structured rather than uniformly distributed across soil profiles (Mo et al., 2024; Epp Schmidt et al., 2022; Shi et al., 2024; Wang et al., 2026). These findings highlight that soil profiles represent not only spatial habitats for microorganisms but also vertically organized ecological compartments through which agricultural practices influence microbial processes. Variations in environmental conditions among soil layers determine the strength of environmental filtering and microbial resource acquisition strategies (Jiang et al., 2025; Mundra et al., 2021), thereby contributing to depth-dependent patterns of microbial community assembly (Song et al., 2022; He et al., 2024). Previous studies have shown that increasing soil depth is generally accompanied by decreases in organic matter inputs, oxygen availability, root-derived resources, and nutrient accessibility, together with stronger physical constraints, resulting in vertically differentiated microbial communities (Wang et al., 2023). Our results further demonstrate that long-term cropping systems modify these intrinsic soil gradients by altering resource distribution and environmental conditions (Mo et al., 2024; Schlatter et al., 2020). Thus, cropping systems may influence microbial community organization indirectly by reshaping environmental heterogeneity and modifying the selective pressures experienced by microorganisms across soil layers (Wang H. T. et al., 2025).

In surface soils (0–20 cm), tobacco–maize rotation provides more diverse plant-derived inputs through crop residues and root-associated carbon inputs (Liu et al., 2023; Wang H. et al., 2025), increasing resource availability and creating more favorable conditions for microbial growth. The increased availability and diversity of substrates may expand microbial niche opportunities, facilitating the coexistence of taxa associated with nitrogen transformation processes, such as nitrifiers and nitrogen-fixing microorganisms (Kong et al., 2025). With increasing soil depth, plant-derived inputs gradually decline, and resource availability becomes increasingly constrained, resulting in stronger environmental selection pressures (Mo et al., 2024). Accordingly, microbial communities in subsurface and deep soils are more strongly associated with resource utilization strategies and environmental adaptation, leading to enhanced vertical differentiation in community composition (Zhu et al., 2025; Gryta et al., 2024). Continuous tobacco cropping further strengthened these depth-related environmental constraints, resulting in greater differentiation of microbial communities among soil layers (Han et al., 2025; Zhang et al., 2026).

From the perspective of microbial community assembly, our findings suggest that cropping systems regulate the balance between resource availability and environmental selection by modifying soil conditions along the vertical profile. Crop rotation maintains relatively stable environmental conditions across soil layers, whereas long-term continuous tobacco cropping intensifies environmental constraints and promotes stronger depth-dependent microbial differentiation. These results indicate that vertical environmental gradients serve as an important ecological linkage between agricultural management and microbial community organization. Moreover, the stratification of environmental filtering provides a basis for subsequent changes in microbial network organization and predicted N-cycling potential. Therefore, considering soil profiles as integrated vertical ecosystems is essential for understanding how long-term cropping practices regulate microbial ecological processes and the sustainability of agricultural soils (Zhao et al., 2019; He et al., 2022). Consistent with previous studies showing that management effects on soil microbiomes generally weaken with increasing depth (Mo et al., 2024; Schmidt et al., 2022), our results further suggest that crop rotation can alleviate depth-related environmental differentiation and maintain relatively continuous environmental conditions throughout the 0–60 cm soil profile.

### 4.2 Depth-related shifts in soil profile community assembly from resource availability to environmental filtering

Based on the vertical environmental gradients observed across the soil profile, this study suggests that the dominant environmental associations shaping microbial community assembly shift progressively with increasing soil depth. Rather than representing isolated layers, soil profiles function as vertically connected ecological systems in which microbial communities experience gradual changes in resource availability and environmental constraints. In surface soils (0–20 cm), bacterial community variation was closely associated with readily available nutrients and plant-derived inputs, reflecting the strong influence of cropping management and resource supply. Tobacco–maize rotation increases organic inputs through diversified crop residues and root-associated carbon inputs (Zhou et al., 2024; Cui et al., 2025; Sun et al., 2025; Wang et al., 2017), potentially improving resource availability and creating favorable conditions for microbial growth and coexistence. Consequently, surface microbial communities under rotation may be more strongly associated with differences in resource supply and nutrient availability.

With increasing depth, the contribution of external resource inputs gradually decreases, whereas environmental constraints become increasingly important. In the 20–40 cm layer, soil pH and microbial biomass parameters (MBC and MBN) exhibited stronger associations with bacterial community composition, suggesting that microbial resource status and biological conditions become increasingly relevant in subsurface environments (He et al., 2023; Muneer et al., 2022). Microbial communities in this layer are likely influenced by both residual resource availability and internal microbial processes, resulting in a transition between surface resource-associated patterns and deeper environmental constraints. In the 40–60 cm layer, the importance of OM, TN, and TP increased, indicating that relatively stable nutrient pools and long-term resource availability were closely associated with community organization (Takele et al., 2025). Under these conditions, taxa with stronger environmental adaptation capacities may become more prevalent, contributing to the distinct community composition observed in deeper soils (Chen S. et al., 2025; Ding et al., 2024; Lei et al., 2026).

The SEM results further indicated that cropping systems modified the relationships among soil properties, bacterial communities, microbial networks, and predicted N-cycling potential. Tobacco–maize rotation was associated with stronger statistical linkages among these components, potentially due to improved soil conditions and more continuous resource inputs throughout the profile (Zong et al., 2024). In contrast, continuous tobacco cropping strengthened the association between microbial communities and environmental constraints, particularly with increasing soil depth, and was accompanied by reduced network connectivity and altered predicted N-cycling potential. These findings suggest that cropping systems regulate microbial community organization by modifying the balance between resource availability and environmental selection along soil profiles.

Overall, our results demonstrate that microbial community assembly is spatially dynamic and closely linked to vertical environmental gradients. Cropping systems do not impose uniform effects across the soil profile; instead, they modify the relative importance of different environmental factors across depths, thereby shaping distinct microbial community patterns and associated predicted functional potential. This depth-dependent perspective extends previous studies showing that subsoil microbial communities respond differently to environmental changes compared with surface soils (Peng et al., 2024) by highlighting the role of cropping-system-induced changes in resource continuity and environmental heterogeneity.

### 4.3 Depth-dependent reorganization of microbial co-occurrence networks under contrasting cropping systems

Microbial co-occurrence network analysis revealed that long-term cropping systems altered microbial interaction patterns and modified network topology across soil profiles. Co-occurrence networks provide a structural representation of potential associations among microbial taxa and can reflect changes in community organization, niche differentiation, and interaction patterns under different environmental conditions (Peng et al., 2024; Kajihara and Hynson, 2024; Xue et al., 2022). Previous studies have shown that environmental changes can reorganize microbial networks by altering species associations, connectivity, and modular structures, thereby influencing the potential stability of microbial communities (Blake et al., 2024; Liu et al., 2025; Clegg and Gross, 2025). However, whether network responses exhibit depth-dependent patterns under long-term agricultural management remains poorly understood (Fujita et al., 2023). In this study, microbial networks under tobacco–maize rotation exhibited distinct topological characteristics compared with continuous tobacco cropping. Although rotation networks did not always show the highest complexity, they maintained relatively high modularity and robustness, suggesting that network organization may depend more on balanced connectivity and compartmentalization than on overall network size (Khan et al., 2025). Increased modularity may allow microbial associations to remain partially independent among different network components while maintaining potential interactions within modules. This pattern indicates a more organized microbial interaction structure under rotation management rather than simply increased network complexity.

In contrast, long-term continuous tobacco cropping resulted in simplified microbial network structures, particularly in the 20–40 cm soil layer. The reductions in network connectivity and inter-module associations suggest weakened microbial associations under intensified environmental constraints (Hu et al., 2026; Xu et al., 2025; Zhang et al., 2024). Such network reorganization may reduce the potential for coordinated microbial interactions and contribute to the altered association between microbial communities and predicted N-cycling potential observed in SEM analysis. However, these network characteristics represent inferred interaction patterns rather than direct measurements of ecological stability. Overall, our findings indicate that microbial network organization provides an important structural link between soil environmental changes and predicted microbial functional potential. Rotation maintains a relatively integrated network architecture by promoting favorable soil conditions and preserving microbial interactions across the profile, whereas continuous cropping promotes network simplification along with increasing depth. These results extend previous findings that rotation can modify microbial network complexity (Kong et al., 2025) by suggesting that network organization, particularly modularity and robustness, may be more informative than complexity alone for interpreting microbial responses to long-term cropping management (Zhou J. B. et al., 2025).

### 4.4 Statistical coupling patterns between microbial communities and predicted N-cycling potential under contrasting cropping systems

Based on the vertical environmental gradients, bacterial community assembly patterns, and microbial network reorganization described above, predicted N-cycling potential exhibited contrasting statistical coupling patterns among cropping systems. Structural equation modeling revealed different association structures among soil properties, bacterial communities, ecological network characteristics, and predicted N-cycling potential, indicating that long-term agricultural management modified the relationships among these ecological components. In tobacco–maize rotation systems, SEM supported stronger positive associations among soil environmental conditions, microbial communities, network characteristics, and predicted N-cycling potential. Continuous crop diversification and residue inputs likely improved resource availability and were associated with the enrichment of taxa potentially involved in nitrogen transformation processes, as well as more connected microbial network structures (Arunrat et al., 2025). Consequently, changes in soil conditions showed stronger statistical associations with predicted N-cycling potential, forming an inferred “soil environment–microbial community–network–predicted function” coupling pattern. These results suggest that rotation maintains stronger ecological connectivity among multiple soil components, which may contribute to the persistence of predicted microbial functional potential.

In unclaimed land, predicted N-cycling potential showed a different association pattern. Although nutrient availability was relatively limited compared with agricultural soils, long-term natural succession established stable relationships between soil environmental conditions and microbial community organization (Zhou Y. J. et al., 2025; Kang et al., 2026). The relatively balanced microbial community structure under natural conditions may allow the maintenance of predicted nitrogen-related functional potential under resource-limited environments. Thus, unclaimed soils represented a weakly coupled but relatively stable statistical association pattern shaped primarily by long-term ecological development rather than continuous external nutrient inputs (Sveen et al., 2025).

In contrast, continuous tobacco cropping exhibited weakened statistical associations among soil properties, microbial communities, network structure, and predicted N-cycling potential. Long-term monocropping intensified environmental constraints and promoted the enrichment of stress-associated taxa, accompanied by reduced network connectivity and altered microbial community organization (Zhou F. F. et al., 2025; Han et al., 2025). Although readily available nutrients accumulated in surface soils, the relationships between resource availability and predicted functional potential became weaker in the SEM model, suggesting that nutrient enrichment alone did not correspond to increased predicted microbial functions. This weakened association may result from multiple interacting processes, including altered microbial community structure, reduced network connectivity, nutrient imbalance, and potential feedback regulation under long-term fertilization regimes (Dong et al., 2025; Li M. et al., 2025). Therefore, the reduced predicted N-cycling potential under continuous cropping should not be attributed solely to microbial network changes but likely reflects the combined influence of resource accumulation, altered microbial organization, and environmental constraints.

It should be noted that the accumulation of AP, AK, and NO₃^−^-N in surface soils under continuous cropping may partly result from long-term fertilizer inputs exceeding crop nutrient uptake. High concentrations of inorganic nitrogen and phosphorus may influence microbial nutrient transformation processes through feedback regulation, potentially reducing the responsiveness of microbial communities to additional nutrient inputs (Hong et al., 2022; Yu et al., 2023). Thus, the observed weakening of statistical coupling under continuous cropping likely represents the integrated outcome of nutrient imbalance, microbial community restructuring, and altered network associations rather than a single controlling mechanism.

Overall, these findings suggest that differences in predicted N-cycling potential among cropping systems are associated not only with resource availability but also with the strength of statistical linkages among soil conditions, microbial communities, and network organization. Rotation was associated with stronger multilevel ecological associations, whereas continuous cropping was associated with weakened coupling patterns under long-term monoculture conditions (Li J. et al., 2025). These results provide a profile-scale framework for understanding how cropping systems reorganize microbial communities and their predicted functional potential through changes in environmental gradients and microbial network structures. However, because this study was based on cross-sectional observations and inferred functional prediction, further experiments integrating functional gene measurements and temporal observations are required to validate these ecological relationships.

### 4.5 An integrated framework linking cropping systems, microbial spatial reorganization, and predicted N-cycling potential across soil profiles

Based on the integrated analyses of soil properties, bacterial communities, microbial networks, and predicted functions, this study proposes a conceptual framework describing how cropping systems regulate microbial spatial reorganization across soil profiles and modify the statistical coupling patterns associated with predicted N-cycling potential (Figure 5). The framework consists of three interconnected processes: environmental gradient modification, depth-dependent microbial community reorganization, and network-mediated functional association. Together, these processes form a cascading pathway from “environmental conditions→ community assembly→network organization→predicted N-cycling potential.”

**Figure 5:**
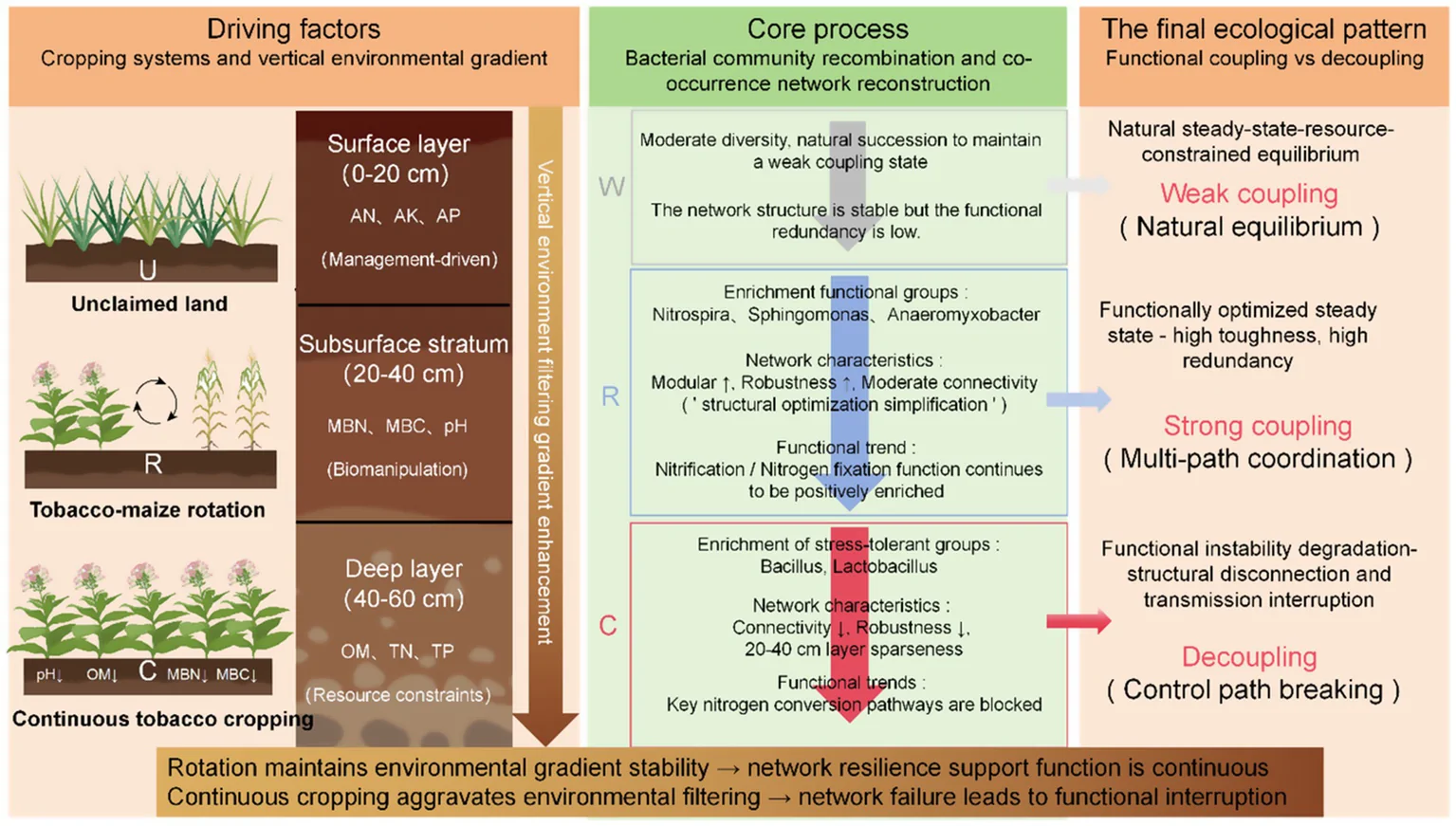
Integrated mechanistic framework illustrating how cropping systems was associated with bacterial community reassembly and N-cycling functional statistical decoupling via reshaping soil profile vertical environmental gradients.

Tobacco–maize rotation was associated with relatively stable environmental conditions throughout the 0–60 cm soil profile, likely due to diversified residue inputs and continuous plant-derived resources. These conditions promoted gradual changes in microbial habitats and supported higher bacterial richness in the 0–40 cm layer, together with the enrichment of taxa potentially involved in nitrogen transformation processes, including Nitrospira, Sphingomonas, and Anaeromyxobacter. In contrast, continuous tobacco cropping altered soil environmental conditions, characterized by surface acidification, reduced organic matter and microbial biomass, and the accumulation of readily available nutrients. This pattern suggests that nutrient accumulation does not necessarily translate into increased microbial functional potential when microbial community organization and environmental conditions are altered.

The integrated analyses further revealed a depth-dependent transition in the environmental factors associated with bacterial community organization. Readily available nutrients (AN, AK, and AP) were more strongly associated with community variation in surface soils, microbial biomass parameters (MBC and MBN) became increasingly important in subsurface soils, and total nutrient pools (OM, TN, and TP) showed stronger associations in deeper soils. Rotation maintained relatively continuous environmental conditions across these depth gradients, whereas continuous cropping intensified differentiation between surface and subsurface environments.

Microbial network analysis further indicated that rotation promoted a more organized interaction structure characterized by higher modularity and robustness, although not necessarily greater network complexity. In contrast, continuous cropping resulted in simplified network structures, particularly in the 20–40 cm layer, with reduced connectivity and altered microbial associations. SEM further suggested stronger statistical coupling among soil properties, bacterial communities, network characteristics, and predicted N-cycling potential under rotation, whereas continuous cropping showed weakened or non-significant associations among these components. These patterns may reflect the combined effects of altered nutrient availability, microbial community restructuring, and changes in network organization rather than a single controlling process.

Overall, this framework highlights that cropping systems regulate soil microbial ecological processes through vertically structured environmental gradients and associated changes in microbial community and network organization. Maintaining relatively continuous environmental conditions and coordinated microbial interactions may contribute to the persistence of predicted N-cycling potential in agricultural soils. This profile-scale framework provides a conceptual basis for understanding how long-term cropping practices reshape microbial ecological processes and offers insights for designing more sustainable agricultural management strategies.

### 4.6 Limitations and future perspectives

This study provides an integrated profile-scale perspective on how long-term cropping systems are associated with changes in soil environmental gradients, bacterial community organization, microbial network structure, and predicted N-cycling potential. The observed depth-dependent patterns are broadly consistent with previous studies showing that the effects of agricultural management on soil microbiomes generally weaken with increasing soil depth (Epp Schmidt et al., 2022; Mo et al., 2024) and that subsoil microbial communities may respond differently, and in some cases more sensitively, to environmental change than surface communities (Peng et al., 2025). Nevertheless, several limitations should be considered when interpreting our findings.

First, soil samples were collected at a single time point during the mature stage of flue-cured tobacco. Therefore, the present study cannot resolve seasonal dynamics, crop developmental effects, or interannual climatic variability in microbial communities and nitrogen-related processes. Moreover, the three treatments represent contrasting long-term management histories rather than sequential stages of ecosystem development. Accordingly, differences among treatments should be interpreted as cross-sectional contrasts rather than temporal or successional trajectories. Long-term repeated sampling across crop developmental stages and multiple years will therefore be necessary to determine the temporal stability of the observed profile-scale patterns.

Second, N-cycling potential was inferred primarily from 16S rRNA gene amplicon sequencing and PICRUSt2-based functional prediction. Although these approaches enable comparative assessment of predicted functional profiles, they do not directly quantify nitrogen transformation rates and cannot distinguish functional gene abundance from transcriptional activity or realized metabolic processes. Consequently, the predicted functional differences reported here should not be interpreted as direct measurements of N-cycling activity. Future studies should integrate functional gene quantification, stable-isotope tracing, metagenomics, metatranscriptomics, and metaproteomics to validate predicted pathways and directly quantify *in situ* nitrogen transformation processes.

Third, this study focused mainly on bacterial communities. Other microbial groups, including archaea, fungi, and protists, were not comprehensively evaluated, despite their potentially important roles in nitrogen transformation and cross-trophic microbial interactions. In particular, archaeal ammonia oxidizers and fungal-mediated nutrient processes may contribute substantially to N cycling in deeper soil layers. Future studies incorporating multi-kingdom community analyses will therefore provide a more complete understanding of microbial interactions and functional organization across soil profiles.

Fourth, the SEM was constructed using cross-sectional data collected at a single sampling time (August 15, 2021). Although the models identified statistical associations among soil properties, bacterial communities, network characteristics, and predicted N-cycling potential that were consistent with the proposed ecological framework, covariance-based SEM using cross-sectional data cannot establish temporal precedence or biochemical causality. The inferred pathways should therefore be interpreted as statistical relationships that generate testable ecological hypotheses rather than evidence of direct causal regulation. Time-series observations, manipulative experiments, and process-based measurements will be required to verify the direction and strength of these proposed relationships.

Fifth, the field experiment was conducted in a representative red-soil tobacco production region of Yunnan Province, where climate, parent material, fertilization regimes, and cropping history may differ substantially from those of other agricultural ecosystems. Therefore, extrapolation of the present findings to other soil types, climatic regions, and cropping systems should be made cautiously. Multi-site validation across contrasting agroecosystems will be necessary to determine the generality of the proposed profile-scale framework.

Future research should combine long-term fixed-site monitoring with high-frequency temporal sampling, multi-omics approaches, direct measurements of nitrogen transformation processes, and controlled manipulation experiments. Integrating climatic variability, management intensity, soil physicochemical properties, and multi-trophic microbial interactions into predictive models may further clarify how soil microbial communities and their functional potential change across both space and time. Such approaches will help determine whether the statistical coupling patterns identified here represent persistent ecological relationships and will provide a more robust basis for developing sustainable cropping strategies that maintain soil microbial functioning across the entire soil profile.

## 5 Conclusion

This study systematically compared soil physicochemical properties, bacterial communities, microbial co-occurrence networks, and predicted N-cycling potential across the 0–60 cm soil profile under three contrasting land-use and cropping systems: unclaimed land, tobacco–maize rotation, and continuous tobacco cropping. The results demonstrate that the effects of cropping systems on soil microbial ecology are strongly depth dependent and are closely associated with vertical changes in soil environmental conditions. Tobacco–maize rotation maintained relatively stable soil nutrient conditions across the profile, increased nutrient availability and bacterial richness in surface and subsurface soils, and enriched several microbial taxa associated with nitrogen transformation processes, including nitrification and nitrogen fixation. These changes were accompanied by higher predicted N-cycling potential across the soil profile. Microbial network analysis further showed that rotation maintained relatively high modularity and robustness, suggesting a more organized microbial interaction structure rather than simply greater network complexity. SEM supported stronger statistical associations among soil properties, bacterial communities, network characteristics, and predicted N-cycling potential under rotation, indicating a more closely coupled “soil environment–microbial community–network–predicted function” relationship. In contrast, continuous tobacco cropping was associated with surface soil acidification, reductions in organic matter and microbial biomass, and marked changes in microbial community composition. Although AP, AK, and NO₃^−^-N accumulated in surface soils, this nutrient enrichment was not accompanied by a corresponding increase in predicted microbial N-cycling potential. Continuous cropping also favored stress-associated and opportunistic taxa and reduced microbial network connectivity and robustness, particularly in the 20–40 cm soil layer. Consistent with these changes, several pathways in the SEM became weak or statistically non-significant, indicating reduced statistical coupling among soil environmental conditions, microbial community organization, network structure, and predicted functional potential. These patterns may reflect the combined effects of nutrient imbalance, altered microbial community structure, network simplification, and feedback regulation under long-term monoculture. Overall, our findings indicate that tobacco–maize rotation is associated with more continuous soil environmental conditions, more organized microbial community and network structures, and stronger statistical coupling with predicted N-cycling potential across the soil profile. By contrast, long-term continuous tobacco cropping disrupts these profile-scale associations and increases vertical differentiation of the soil microbial ecosystem. These results provide a profile-scale framework for understanding how cropping systems reshape soil microbial organization and predicted nitrogen-cycling potential, and highlight the importance of considering subsurface soil layers when designing sustainable agricultural management strategies.

## Data Availability

The datasets presented in this study can be found in online repositories. The names of the repository/repositories and accession number(s) can be found in the article/Supplementary material.

## Footnotes

1
https://www.omicshare.com/tools/home/task/index.html

2
https://cloud.metware.cn
