# Supplementary material for: Rotation system reshapes soil profile environmental gradients to reorganize bacterial communities and decouple potential nitrogen cycling functions
Source: Front Microbiol. 2026 Oct 14;17:1979930. doi: 10.3389/fmicb.2026.1979930 (PMC13713252; doi:10.3389/fmicb.2026.1979930)
Supplement: Supplementary file 1 [file Supplementary_file_1.docx]

***Supplementary Material***

**Rotation system reshape soil profile environmental gradients to reorganize bacterial communities and decouple potential nitrogen cycling functions**

Wenping Zhang^1,2^, Qinyue Chen^1,2^, Yunguo Li^3^, Yan Chen^4^, Guoyi Yuan^1,2^, Yu Du^1,2^, Bing Wang^1,2^, Xinran Sun^1,2^, Nuo Shi^1,2^, Xuanquan Zhu^1,2^, Meng Jia^1,2^, Yuxiang Bai ^1,2^, Xiaopeng Deng^5*^, Peng Zhou^1,2*^, Ge Wang^1,2*^, Na Wang^1,2*^

^1^College of Tobacco Science, Yunnan Agricultural University, Kunming, Yunnan, 650201, China

^2^The Key Laboratory of Agricultural Microbiome of Yunnan Province, Kunming, Yunnan, 650201, China

^3^Yuxi Tobacco Company Yuanjiang County Branch, Yuxi, Yunnan, 653300, China

^4^Honghe Hani and Yi Autonomous Prefecture Academy of Science and Technology, Honghe, Yunnan, 661199, China

^5^Yunnan Provincial Institute of Tobacco and Agricultural Sciences, Yunnan, 650000, China

Figure S1. Differentially abundant species and nitrogen cycling functional characteristics of soil bacterial communities. A-C: LEfSe analysis of differentially abundant bacterial taxa across soil depth layers (0-20, 20-40, 40-60 cm); D: Heatmap showing the abundance of nitrogen cycling-related functions in soils under different depth layers and treatments. U: Unclaimed land; R: Tobacco-maize rotation; C: Continuous tobacco cropping.
